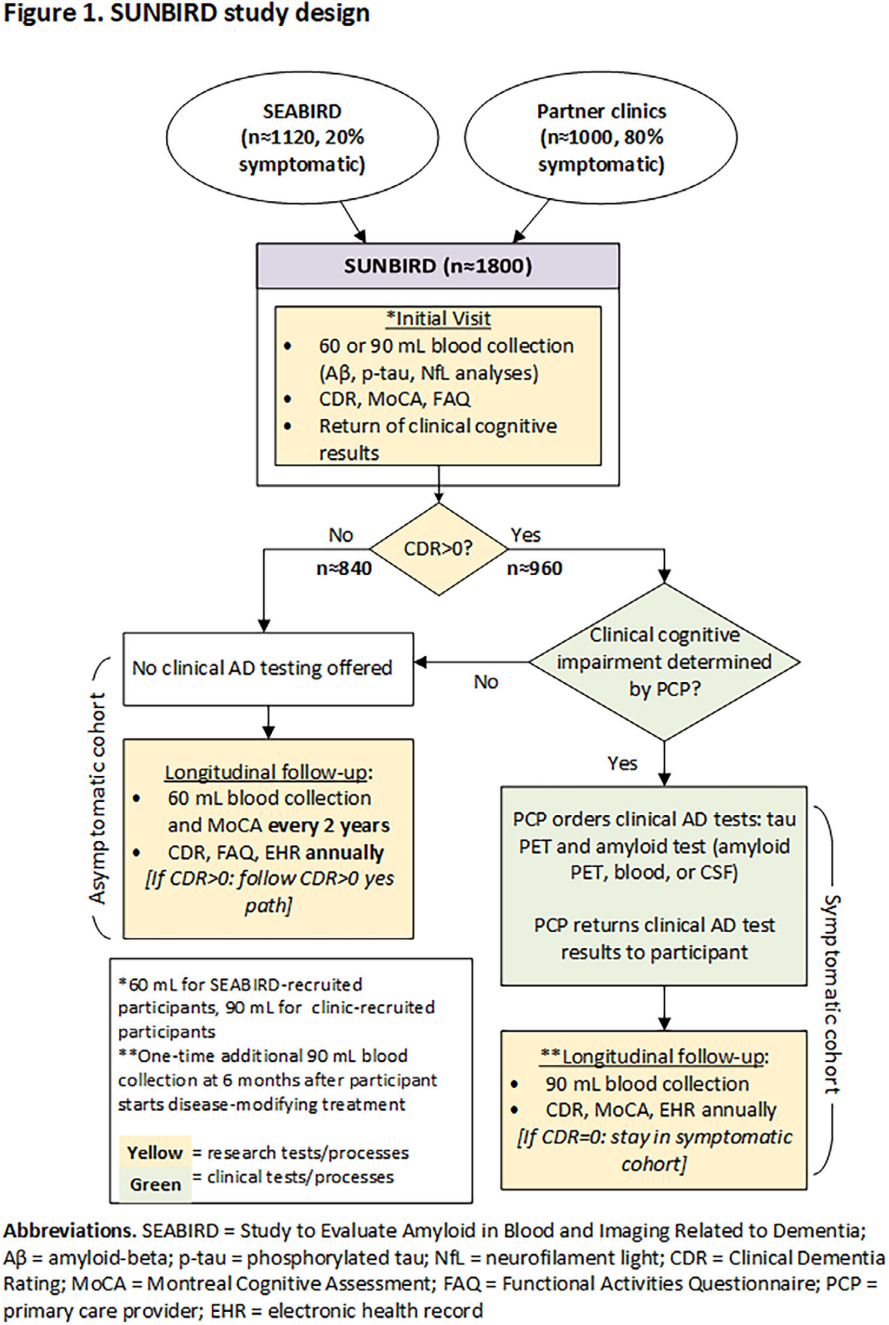# Evaluating Alzheimer's disease blood biomarkers in a real‐world primary care clinical population: The study design of SUNBIRD

**DOI:** 10.1002/alz70856_103300

**Published:** 2025-12-25

**Authors:** Melody Li, Lisa Soke, Nupur Ghoshal, David B. Carr, Randall J. Bateman

**Affiliations:** ^1^ Washington University School of Medicine in St. Louis, St. Louis, MO, USA; ^2^ The Tracy Family SILQ Center, St. Louis, MO, USA; ^3^ Washington University School of Medicine, St. Louis, MO, USA

## Abstract

**Background:**

With the implementation of anti‐amyloid therapies to slow the progression of Alzheimer's disease (AD), early and accurate diagnosis is critical to identify individuals who may benefit from treatment. Blood tests to aid in diagnosis are clinically available but have not been tested in primary care settings for the impact on diagnosis and treatment or prediction of clinical and cognitive decline. Questions remain on how to optimally use different blood biomarkers (e.g., amyloid‐beta, phospho‐tau, and MTBR‐tau) and effects of comorbidities, race, sex, education, and other factors. To address these questions, the Study to Understand Novel Biomarkers in Researching Dementia (SUNBIRD) was launched in August 2024 to longitudinally follow approximately 2000 participants in the Saint Louis, Missouri, USA area.

**Method:**

SUNBIRD participants are recruited from a recently completed, diverse community‐based cohort (SEABIRD: *n* = 1120, 80% asymptomatic) and a new clinic‐based cohort focused on primary care and community neurology clinics (planned *n* = 1000, 80% symptomatic) (Figure 1). All participants complete a research blood collection for amyloid, tau, and neurofilament biomarkers, Clinical Dementia Rating® (CDR), Montreal Cognitive Assessment, Functional Activities Questionnaire, and a survey about their study experience. Clinical cognitive test results are shared with the participant's primary care provider (PCP). For symptomatic participants, PCPs have the option to order a clinical tau PET and their choice of an amyloid test (amyloid PET, blood, or cerebrospinal fluid). All participants are followed longitudinally with research blood collections, clinical cognitive assessments, and electronic health record data extraction including clinical and cognitive tests, diagnoses, comorbidities, and medications.

**Result:**

Enrollment for the study is well underway with participants recruited from SEABIRD and partner clinics. Participant survey results indicate that to date the study is well accepted. The design, implementation, and enrollment progress of the prior SEABIRD and ongoing SUNBIRD studies will be reviewed with outcomes of clinical testing and referrals.

**Conclusion:**

Enrollment of participants in this real‐world study and engagement of PCPs and community neurologists in the clinical AD diagnostic process are feasible. Further work will address ongoing questions about the validity and clinical use of blood biomarkers in the clinic in the diagnosis and treatment of AD.